# Clinical course of patients with rheumatoid arthritis who continue or discontinue biologic therapy after hospitalization for infection: a retrospective observational study

**DOI:** 10.1186/s13075-022-02820-y

**Published:** 2022-06-01

**Authors:** Yusuke Kashiwado, Chikako Kiyohara, Yasutaka Kimoto, Shuji Nagano, Takuya Sawabe, Kensuke Oryoji, Shinichi Mizuki, Hiroaki Nishizaka, Seiji Yoshizawa, Shigeru Yoshizawa, Tomomi Tsuru, Yasushi Inoue, Naoyasu Ueda, Shun-ichiro Ota, Yasuo Suenaga, Tomoya Miyamura, Yoshifumi Tada, Hiroaki Niiro, Koichi Akashi, Takahiko Horiuchi

**Affiliations:** 1 Department of Internal Medicine, Kyushu University Beppu Hospital, 4546 Tsurumibaru, Beppu, Oita 874-0838 Japan; 2 Department of Preventive Medicine, Graduate School of Medical Sciences, Kyushu University, Fukuoka, Japan; 3 Department of Rheumatology, Aso Iizuka Hospital, Iizuka, Japan; 4 Department of Rheumatology, Hiroshima Red Cross Hospital & Atomic-bomb Survivors Hospital, Hiroshima, Japan; 5 The Center for Rheumatic Diseases, Matsuyama Red Cross Hospital, Matsuyama, Japan; 6 Department of Rheumatology, Kitakyushu Municipal Medical Center, Kitakyushu, Japan; 7 Department of Rheumatology, Hamanomachi Hospital, Fukuoka, Japan; 8 Department of Rheumatology, National Hospital Organization Fukuoka Hospital, Fukuoka, Japan; 9 Department of Rheumatology, Med.Co. LTA PS Clinic, Fukuoka, Japan; 10 Department of Rheumatology, Japanese Red Cross Fukuoka Hospital, Fukuoka, Japan; 11 Department of Rheumatology and Infection, Miyazaki Prefectural Miyazaki Hospital, Miyazaki, Japan; 12 Department of Rheumatology, Internal medicine and connective tissue disorders, Shimonoseki City Hospital, Shimonoseki, Japan; 13 Department of Rheumatology, Beppu Medical Center, NHO, Beppu, Japan; 14 Department of Internal Medicine and Rheumatology, National Hospital Organization Kyushu Medical Center, Fukuoka, Japan; 15 Department of Rheumatology, Saga University Hospital, Saga, Japan; 16 Department of Medical Education, Graduate School of Medical Sciences, Kyushu University, Fukuoka, Japan; 17 Department of Medicine and Biosystemic Science, Graduate School of Medical Sciences, Kyushu University, Fukuoka, Japan

**Keywords:** Rheumatoid arthritis, Infection, Biological therapy, Antirheumatic agents

## Abstract

**Background:**

To analyse the subsequent clinical course of patients with rheumatoid arthritis (RA) who either continued or discontinued biologic agents after hospitalization for infections.

**Methods:**

We retrospectively reviewed the clinical records of 230 RA patients with 307 hospitalizations for infections under biologic therapy between September 2008 and May 2014 in 15 institutions for up to 18 months after discharge. The risks of RA flares and subsequent hospitalizations for infections from 61 days to 18 months after discharge were evaluated.

**Results:**

Survival analyses indicated that patients who continued biologic therapy had a significantly lower risk of RA flares (31.4% vs. 60.6%, *P* < 0.01) and a slightly lower risk of subsequent infections (28.7% vs. 34.5%, *P* = 0.37). Multivariate analysis showed that discontinuation of biologic therapy, diabetes, and a history of hospitalization for infection under biologic therapy were associated with RA flares. Oral steroid therapy equivalent to prednisolone 5 mg/day or more and chronic renal dysfunction were independent risk factors for subsequent hospitalizations for infections.

**Conclusions:**

Discontinuation of biologic therapy after hospitalization for infections may result in RA flares. Continuation of biologic therapy is preferable, particularly in patients without immunodeficiency.

**Supplementary Information:**

The online version contains supplementary material available at 10.1186/s13075-022-02820-y.

## Background

Over the last decade, biologic disease-modifying anti-rheumatic drugs (DMARDs) have been broadly used in patients with rheumatoid arthritis (RA) [1]. These excellent therapeutic agents improve clinical symptoms, physical function, and quality of life and are recommended in patients with moderate to severe RA [2, 3]. However, because biological DMARDs suppress cytokines and the function of cells associated with immunological defence mechanisms, the most frequent serious adverse event associated with this treatment is a severe infection. Some patients under biologic therapy need hospitalization for infections every year [4, 5].

Once patients have recovered from their infection and are to be discharged, a decision has to be made whether they should continue biologic therapy. It would assist physicians to understand the risk of RA exacerbation if biologic agents are discontinued as well as the risk of subsequent severe infections if they are continued. However, to our knowledge, only two retrospective studies have reported on the risk of infections in RA patients receiving biologic agents after having been hospitalized with infections [6, 7]. These studies focused on RA patients who had been hospitalized with infections who were under anti-tumour necrosis factor (anti-TNF) therapy and analysed the risk of subsequent hospitalizations because of infections under continued therapy. These studies did not evaluate the impact of discontinuation of biological agents on RA exacerbations.

The aim of the present study was to evaluate the benefits and risks of continued versus discontinued biologic therapy with regard to RA flares and subsequent hospitalization for infections in patients who had previously been hospitalized with an infection that occurred under biologic therapy.

## Methods

### Participants

This retrospective observational study was conducted in RA patients hospitalized with a diagnosis of infectious disease under biologic therapy from September 2008 to May 2014 in 15 rheumatological institutions located in Southwest Japan. Three of the institutions were university hospitals. The others were independent hospitals affiliated with Kyushu University. Hospitalizations for infections were identified by the diagnostic name in discharge summaries of RA patients who were receiving biological DMARDs in the outpatient departments of these institutions and were hospitalized during the period. The patients were divided into two groups according to continuation or discontinuation of biologic therapy after discharge. Continuation of biologic therapy was defined as the administration of any biologic DMARDs within 60 days after discharge because the 56-day maintenance interval of infliximab is the longest among the biologic DMARDs approved in Japan.

**Fig. 1:**
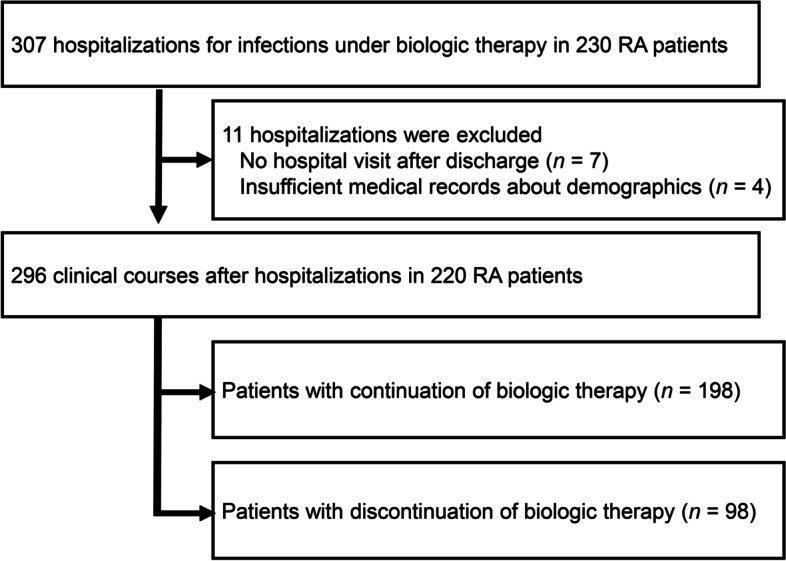
Study flow chart of 230 patients with rheumatoid arthritis hospitalized with infection

All the patients in this study were Japanese. We identified 230 patients with 307 hospitalizations for infections. Eleven hospitalizations were excluded because of no hospital visit after discharge (*n* = 7) or insufficient medical records (*n* = 4). Eventually, patients’ demographics were sufficiently documented in 296 cases. One hundred ninety-eight patients continued biologic DMARDs, and 98 discontinued (Fig. 1). The mean age of patients was 64.7 (63.4–65.9), and 216 (73.0 %) of them were female (Table 1).

**Table 1:** Baseline characteristics at discharge of 307 rheumatoid arthritis patients hospitalized with infection

	Patients (*n* = 296)		Patients with continuation of biologic therapy (*n* = 198)		Patients with discontinuation of biologic therapy (*n* = 98)		*P*-value*
Age (years), mean (95% CI)	64.7	(63.4–65.9)	63.3	(61.7–64.9)	67.4	(65.3–69.4)	< 0.01
Gender (female), *n* (%)	216	(73.0)	150	(75.8)	66	(67.4)	0.13
Disease duration (years), mean (95% CI)	13.6	(12.2-14.9)	13.4	(11.8–15.1)	13.8	(11.4–16.2)	0.82
≥ 10 years, *n* (%)	155	(52.4)	100	(50.5)	55	(56.1)	0.36
Stage III or IV, *n* (%)	208	(70.3)	138	(69.7)	70	(71.4)	0.76
Class III or IV, *n* (%)	80	(27.0)	41	(20.7)	39	(39.8)	< 0.01
RF positive, *n* (%)	258	(87.2)	174	(87.9)	84	(85.7)	0.6
ACPA positive, *n* (%)	143/182	(78.6)	90/113	(79.7)	53/69	(76.8)	0.65
Chronic lung disease, *n* (%)	137	(46.3)	88	(46.3)	49	(50.0)	0.37
Chronic renal dysfunction (eGFR < 60 ml/min), *n* (%)	105	(35.5)	64	(32.3)	41	(41.8)	0.11
Diabetes, *n* (%)	84	(28.4)	52	(26.3)	32	(32.7)	0.25
Past history of hospitalization for infection under prior biologic therapy, *n* (%)	77	(26.0)	56	(28.3)	21	(21.4)	0.2
RA treatment at discharge							
Biologics use, *n* (%)	198	(66.9)	198	(100.0)	0	(0)	-
MTX use, *n* (%)	132	(44.6)	96	(48.5)	36	(36.7)	0.05
MTX dose (mg/week), mean (95%CI)	3.5	(3.0–4.0)	3.8	(3.2-4.5)	2.7	(1.9–3.4)	0.03
Oral steroid use, *n* (%)	245	(82.8)	159	(80.3)	86	(87.8)	0.11
Equivalent dose to > 5 mg/day prednisolone, *n* (%)	143	(48.3)	81	(40.9)	62	(63.3)	< 0.01
Oral steroid dose (mg/day), mean (95%CI)	6.1	(5.6–6.7)	5.3	(4.7–5.8)	7.9	(6.7–9.0)	< 0.01
Other DMARDs use, *n* (%)	79	(26.7)	49	(24.8)	30	(30.6)	0.28
Observation outcomes within 18 months after discharge							
RA flare^a^	131/275	(47.6)	69/189	(36.5)	62/86	(72.1)	< 0.01
Subsequent hospitalization for infection^b^	110/284	(38.7)	67/191	(35.1)	43/93	(46.2)	0.07
Death	19/287	(6.6)	6/192	(3.1)	13/95	(13.7)	< 0.01

*Abbreviations*: *CI* confidence interval, *Stage* Steinbrocker stage, *Class* Steinbrocker class, *RF* rheumatoid factor, *ACPA* anti-citrullinated peptide antibody, *MTX* methotrexate, *DMARDs* disease-modifying anti-rheumatic drugs

*Calculated by Student’s *t*-test or *χ*^2^ test

^a^21 patients dead or lost to follow-up before RA flares were excluded

^b^12 patients dead or lost to follow-up before subsequent hospitalizations for infection were excluded

The information regarding this study was announced on the hospital website, and patients were given the opportunity to opt-out. This study was performed in accordance with the Declaration of Helsinki and was approved by the ethics committee of Kyushu University Hospital (approval number 28-255).

### Data collection

We reviewed the inpatient and outpatient records of patients up to 18 months after their hospitalization. Twenty-eight patients died or were lost to follow-up within 18 months, and the others were alive and visited the hospital. The clinical records at discharge included age, sex, RA characteristics (disease duration, Steinbrocker classification, positivity for rheumatoid factor (RF) and/or anti-citrullinated peptide antibody (ACPA)), RA medication (corticosteroid dosage (mg/day), methotrexate dosage (mg/week), biologic DMARDs, other), comorbidities (diabetes, lung disease, and chronic renal dysfunction), and a history of hospitalization because of an infection under biologic therapy. Diabetes was defined by usage of insulin or oral antidiabetic drugs during hospitalization. Lung disease included chronic bronchitis, bronchiectasis, and interstitial pneumonia confirmed through imaging tests. Chronic renal dysfunction was defined as an estimated glomerular filtration rate (eGFR) < 60 ml/min at discharge.

The follow-up data on subsequent hospitalizations for infection, RA flares, and death were obtained from outpatient clinical records. An RA flare was defined as an increase or initiation of conventional synthetic DMARDs or oral steroids compared to the medication at discharge or a change in dosage or new administration of biologic DMARDs more than 60 days after discharge. When RA activity was stable after discharge, the physicians would adopt two different policies of corticosteroids or conventional synthetic DMARDs usage, i.e. either continuation of the same dose or reduction. If RA was flared in the course of reduction and RA treatment was intensified, it does not reflect the ‘real flare’ caused by continuation or discontinuation of biologic therapy but was dependent on the reduction in the dose of the anti-RA drug. Therefore, we decided to define flares as increases of these medications compared to those at the discharge.

### Statistical analysis

We compared the means and proportions between the two groups with either Student’s *t*-test or *χ*^2^-test. The Cox proportional hazards regression models were used to estimate hazard ratios (HRs) and 95% confidence intervals (CIs) for the association between demographics at discharge and RA flares or subsequent hospitalization for infection between 61 days and 18 months after discharge. Death or loss to follow-up was treated as censoring. The Kaplan-Meier method was used to estimate subsequent hospitalizations for infection and the log-rank method to compare the curves. All *P*-values were two-sided and considered statistically significant if they were < 0.05. All statistical analyses were performed using the programme Stata Statistical Software: Release 14 (StataCorp, College Station, TX, USA).

## Results

### Clinical course after discharge

Patients’ characteristics at discharge and the clinical course up to 18 months after discharge are shown in Table 1. The crude rates of RA flares, subsequent hospitalization for infections, and death within 18 months in all patients were 47.6%, 38.7%, and 6.6%, respectively. The rate of RA flares was significantly higher in patients who discontinued biological therapy compared to those who continued (36.5% vs. 72.1%; *P* < 0.01). The same applied for death (3.1% vs. 13.7%; *P* < 0.01). The Cox proportional hazard model adjusted for age and sex showed that oral steroid use and Steinbrocker class III or IV were positively, but biologics and MTX use was negatively associated with mortality (Supplementary Table S1).

Anti-TNF inhibitor treatment accounted for 75.6 % and 72.3 % of biologic DMARDs before and after hospitalization, respectively (Supplementary Table 2). Changes in biologic DMARDs at discharge were seen only in 16 cases (8.1%) [data not shown]. There were no statistically significant difference of RA flares and subsequent hospitalization for infection among biologic treatment groups (Supplementary Figures S1 and S2).

### RA flares

**Fig. 2:**
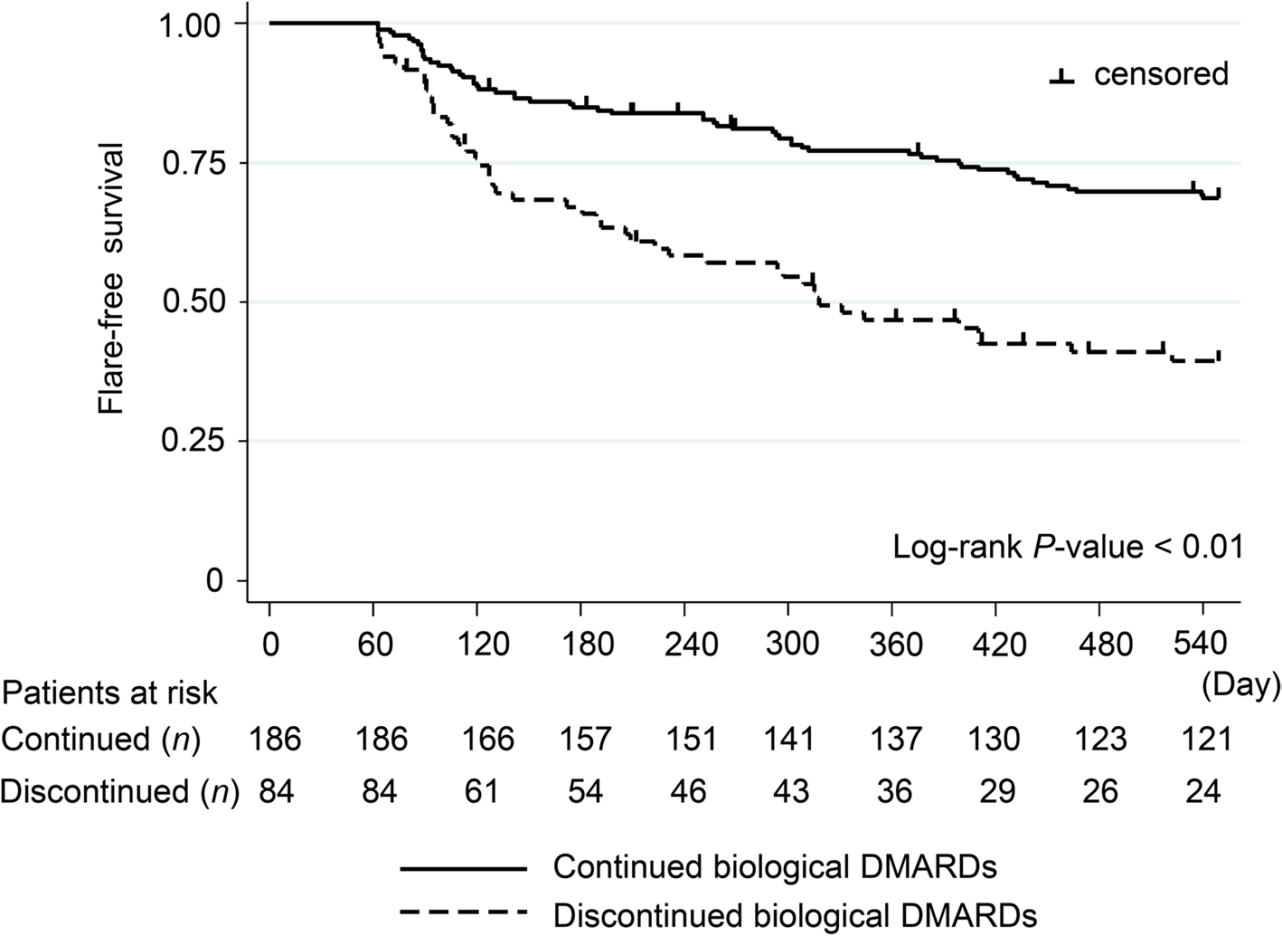
Kaplan-Meier curves of RA flare-free survival. The curves compare patients with and without continuation of biologic disease-modifying anti-rheumatic drugs (DMARDs) after hospitalization for infection. RA flares within 60 days after discharge were excluded from the analysis

One hundred thirty-one RA flares were seen in 296 patients. Two hundred seventy patients were analysed for RA flare, with the exception of 26 patients whose RA flares occurred within 60 days after discharge. The probability of RA flares was significantly lower in patients who continued biologic DMARDs at discharge than in those who discontinued them (31.4% vs. 60.6%; *P* < 0.01, log-rank test; Fig. 2). In the multivariate analysis, discontinuation of biologic DMARDs (HR = 2.75, 95% CI = 1.81–4.18; *P* < 0.01), diabetes (HR = 0.43, 95% CI = 0.26–0.71; *P* < 0.01), and a history of hospitalization for infection prior to biological therapy (HR = 1.74, 95% CI = 1.14–2.67; *P* = 0.01) were significantly associated with RA flares (Table 2).

**Table 2:** Cox multivariate analysis for risk of flares in rheumatoid arthritis patients after hospitalization for infection

	Crude		Model 1^a^		Model 2^b^	
	HR (95% CI)	*P*-value	HR (95% CI)	*P*-value	HR (95% CI)	*P*-value
Age (years)	0.99 (0.98–1.01)	0.53	–	–	–	–
Gender; female vs. male	0.86 (0.57–1.32)	0.50	–	–	–	–
Disease duration (years)	0.99 (0.97–1.01)	0.27	0.99 (0.97–1.01)	0.35	–	–
≥ 10 years vs. < 10 years	1.02 (0.69–1.49)	0.93	1.05 (0.71–1.54)	0.82	–	–
Stage III or IV vs. I or II	1.31 (0.84–2.04)	0.23	1.31 (0.84–2.04)	0.23	–	–
Class III or IV vs. I or II	1.35 (0.90–2.03)	0.15	1.39 (0.92–2.09)	0.12	0.96 (0.62–1.50)	0.87
RF positive vs. negative	0.87 (0.50–1.50)	0.61	0.83 (0.48–1.45)	0.51	–	–
ACPA positive vs. negative	1.04 (0.58–1.87)	0.89	1.03 (0.57–1.85)	0.92	–	–
Discontinuation of biologic therapy	2.52 (1.72–3.71)	< 0.01	2.61 (1.76–3.85)	< 0.01	2.75 (1.81–4.18)	< 0.01
MTX use vs. no use	1.23 (0.84–1.81)	0.29	1.23 (0.82–1.83)	0.31	–	–
MTX dose (mg/week)	1.03 (0.99–1.07)	0.20	1.03 (0.98–1.07)	0.23	–	–
Oral steroid, > 5 mg/day vs. ≤5 mg/day	1.45 (0.98–2.12)	0.06	1.42 (0.96–2.12)	0.08	1.39 (0.92–2.10)	0.12
Oral steroid dose (mg/day)	1.02 (0.98–1.06)	0.40	1.01 (0.97–1.06)	0.51	–	–
Other DMARDs use vs. non use	1.29 (0.86–1.95)	0.23	1.32 (0.87–2.00)	0.19	1.25 (0.82–1.92)	0.30
Chronic lung disease	0.94 (0.64–1.38)	0.75	0.93 (0.60–1.42)	0.73	–	–
Chronic renal dysfunction (eGFR < 60 ml/min)	0.78 (0.51–1.20)	0.25	0.80 (0.50–1.29)	0.36	–	–
Diabetes	0.55 (0.33–0.88)	0.01	0.53 (0.50–1.19)	0.01	0.43 (0.26–0.71)	< 0.01
A history of hospital-acquired infection under biologic therapy	1.45 (0.96–2.20)	0.08	1.48 (0.97–2.25)	0.07	1.74 (1.14–2.67)	0.01

*Abbreviations*: *CI* confidence interval, *HR* hazard ratio, *Stage* Steinbrocker stage, *Class* Steinbrocker class, *RF* rheumatoid factor, *ACPA* anti-citrullinated peptide antibody, *MTX* methotrexate, *DMARDs* disease-modifying anti-rheumatic drugs

^a^Adjusted for age and sex

^b^Adjusted for variables with *P*-value < 0.20 in addition to model 1 variables

### Subsequent hospitalization for infection

**Fig. 3:**
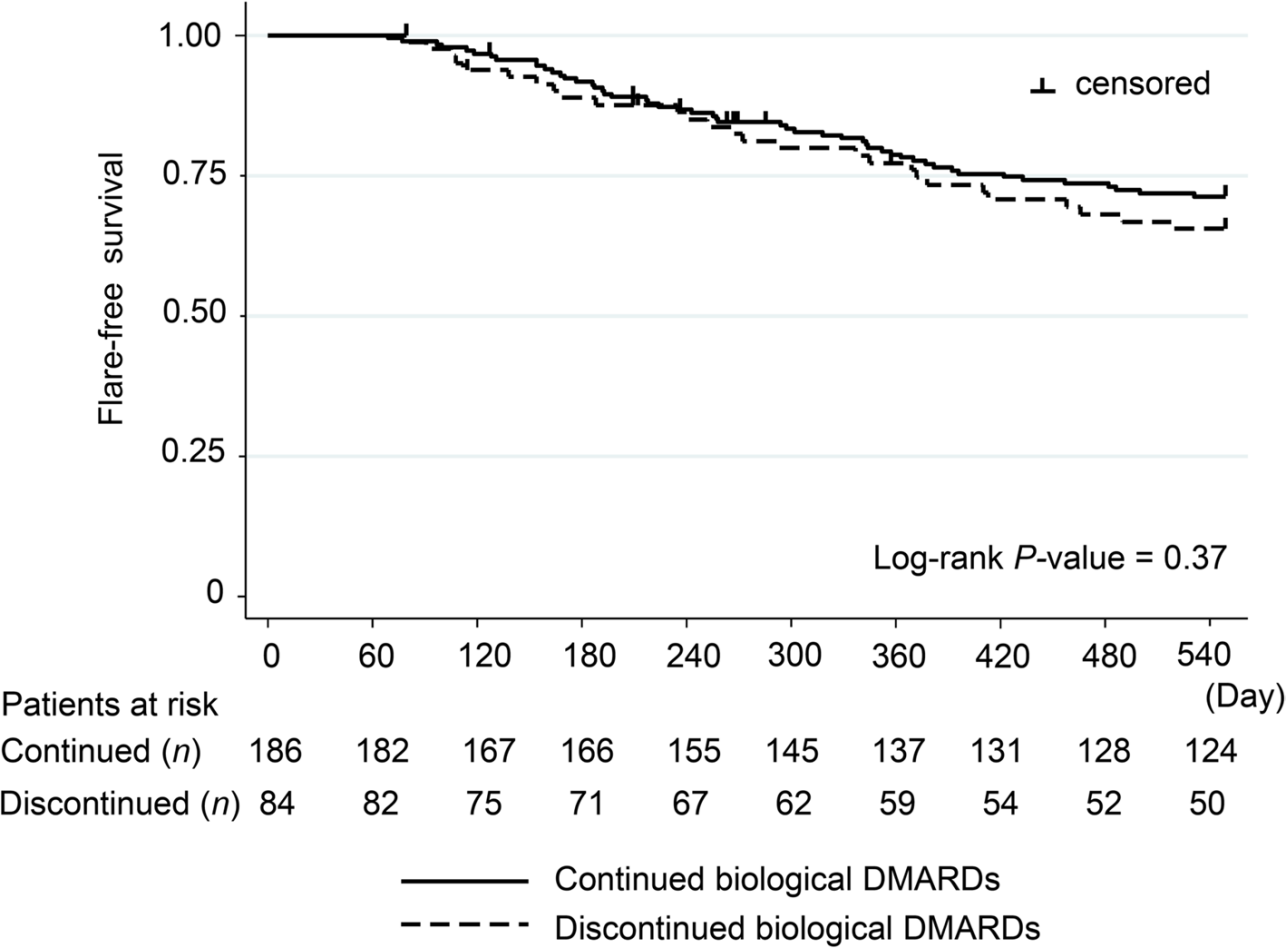
Kaplan-Meier curves of hospitalization for infection-free survival. The curves compare patients with or without continuation of biologic disease-modifying anti-rheumatic drugs (DMARDs) after hospitalization for infection. All hospitalizations for infections within 60 days after discharge were excluded from analysis

One hundred and ten subsequent hospitalizations for infections were seen in 296 patients. Two hundred sixty-four patients for subsequent hospitalized infection were analysed, with the exception of 32 cases hospitalized for infection within 60 days after discharge. The probability of subsequent hospitalization for infections was not significantly different between RA patients who continued biologic DMARDs at discharge and those who discontinued them (28.7% vs. 34.5%, *P* = 0.37, log-rank test; Fig. 3). Multivariate analysis showed that oral steroids in equivalent doses to prednisolone 5 mg/day or more (HR = 2.25, 95% CI = 1.37–3.67; *P* < 0.01) and chronic renal dysfunction (HR = 1.69, 95% CI = 1.01–2.83; *P* = 0.04) were significantly associated with subsequent hospitalization for infections, while continuation of biological DMARDs was not (Table 3).

**Table 3:** Cox multivariate analysis for risk of subsequent hospitalization for infection in rheumatoid arthritis patients

	Crude		Model 1^a^		Model 2^b^	
	HR (95% CI)	*P*-value	HR (95% CI)	*P*-value	HR (95% CI)	*P*-value
Age (years)	1.00 (0.99–1.02)	0.63	–	–	–	–
Gender; female vs. male	0.86 (0.56–1.42)	0.56	–	–	–	–
Disease duration (years)	1.00 (0.99–1.02)	0.65	1.00 (0.99–1.02)	0.67	–	–
≥ 10 years vs. < 10 years	1.03 (0.67–1.62)	0.86	1.03 (0.66–1.62)	0.90	–	–
Stage III or IV vs. I or II	1.05 (0.64–1.72)	0.85	1.05 (0.64–1.72)	0.90	–	–
Class III or IV vs. I or II	1.36 (0.84–2.19)	0.21	1.37 (0.85–2.23)	0.20	–	–
RF positive vs. negative	1.57 (0.72–3.40)	0.26	1.57 (0.72–3.44)	0.26	–	–
ACPA positive vs. negative	1.47 (0.65–3.28)	0.35	1.47 (0.65–3.31)	0.36	–	–
Biologics use vs. no use	0.81 (0.51–1.29)	0.37	0.83 (0.51–1.33)	0.43	–	–
MTX use vs. no use	0.67 (0.42–1.06)	0.09	0.68 (0.42–1.09)	0.11	0.96 (0.57–1.61)	0.88
MTX dose (mg/week)	0.98 (0.92–1.03)	0.38	0.98 (0.92–1.04)	0.44	–	–
Oral steroid, > 5 mg/day vs. ≤5 mg/day	2.45 (1.54–3.89)	< 0.01	2.50 (1.56–4.01)	< 0.01	2.25 (1.37–3.67)	< 0.01
Oral steroid dose (mg/day)	1.05 (1.01–1.08)	0.02	1.04 (1.01–1.09)	0.02	–	–
Other DMARDs use vs. no use	1.40 (0.87–2.26)	0.16	1.38 (0.86–2.23)	0.18	1.13 (0.68–1.88)	0.64
Chronic lung disease	1.44 (0.92–2.24)	0.11	1.42 (0.88–2.29)	0.15	1.26 (0.77–2.06)	0.36
Chronic renal dysfunction (eGFR < 60 ml/min)	1.79 (1.15–2.80)	0.01	1.96 (1.19–3.24)	< 0.01	1.69 (1.01–2.83)	0.04
Diabetes	1.42 (0.89–2.28)	0.14	1.39 (0.86–2.25)	0.18	1.09 (0.66–1.81)	0.73
A history of hospital-acquired infection in the prior biologic therapy	1.38 (0.85–2.25)	0.19	1.37 (0.84–2.23)	0.21	-	-

*Abbreviations*: *CI* confidence interval, *HR* hazard ratio, *Stage* Steinbrocker stage, *Class* Steinbrocker class, *RF* rheumatoid factor, *ACPA* anti-citrullinated peptide antibody, *MTX* methotrexate, *DMARDs* disease-modifying anti-rheumatic drugs

^a^Adjusted for age and sex

^b^Adjusted for variables with *P*-value < 0.20 in addition to model 1 variables

## Discussion

This retrospective study evaluated the clinical course of RA patients who either continued or discontinued the use of biologic agents after having been hospitalized for infections. Our findings compared the rate and risk factors of RA flares and subsequent hospitalization for infections between these patients. The patients who discontinued biologic therapy experienced RA flares more frequently than those who continued and experienced subsequent hospitalization for infections at the same frequency.

The effects of discontinuation of biologic therapy were studied in many randomized controlled trials in patients who were in remission or showed low disease activity. The rate of RA flares during the first year after discontinuation ranged from 19 to 87% in these studies [8–14]. Following these results, the European League Against Rheumatism [2] and American College of Rheumatology [3] recommended reducing biological DMARDs rather than discontinuation for RA patients in sustained remission. The rate of RA flares in the patients who discontinued biologic therapy in our study was 72.1%. Therefore, discontinuation of biologic therapy just because a patient needs hospitalization for infection does not seem desirable from the perspective of disease control.

Switching to another biologic DMARD or reducing the dose of biologic DMARDs instead of stopping them entirely may be beneficial to both minimize the risk of subsequent infection and prevent RA flares. Yun et al. [6], in their retrospective study, showed that patients who switched from a TNF-inhibitor to abatacept or etanercept after hospitalization for an infection experienced less subsequent hospitalizations for infections than those who continued the TNF-inhibitor. Abatacept [15] and etanercept [16–18] were reported to be associated with a lower risk of severe infections than other biologic DMARDs in a general population of RA patients. A statistical analysis of patients switching biologic DMARDs could not be performed, because only 16 patients changed their biologic DMARD after hospitalization. We showed that such switching was not common in our Japanese RA population.

Among RA patients in remission, dose reduction of biologic DMARDs was shown to be less likely to cause exacerbation of RA than stopping biologic DMARDs altogether [8, 11, 19, 20]. A systematic review showed that standard- and high-dose biologic DMARDs were associated with an increase in serious infections compared to conventional synthetic DMARDs in RA, while low-dose biologic DMARDs were not [21].

The continuation of biologic therapy was not significantly associated with subsequent severe infections in the present study. Accortt et al. [7] showed a similar result in patients receiving TNF-inhibitor treatment for rheumatic conditions, including RA, psoriatic arthritis, ankylosing spondylitis, and psoriasis. Yun et al. [6] also found that the risk of subsequent infection was estimated by using demographics, co-morbidities, and concurrent medications in addition to the kind of biologics used. They found that the crude rate of subsequent infection was lower in patients who discontinued biologics than in those who continued them, but they did not analyse whether the continuation of biologic agents itself was a risk for subsequent infection [6]. These retrospective results and those of our study imply that the individual degree of a patient’s immunodeficiency is the main determinant of the risk of subsequent serious infections among immunocompromised patients hospitalized with infection, even if biological therapy was one of the risk factors of severe infection in a general RA population [4].

Our analysis showed that absence of diabetes and a history of hospitalization for infection under prior biological therapy were associated with high risk of RA flares. These factors were not reported to be associated with RA flares in RA patients receiving biologic therapy [22] or not [23]. Daïen et al. [24] showed that the presence of diabetes at RA diagnosis was a risk factor of poor outcomes. Because RA flares were defined as intensification of RA treatment in our study, the physician’s view on avoiding treatment intensification because the patient had been taking several medications for diabetes, which may have influenced our results.

There are limitations to our study because of its retrospective nature. The data analysed may not be complete, accurate, or consistently measured among patients. Additionally, intensification of RA treatment was used as a surrogate marker for RA flares because more accurate markers of disease activity such as the Disease Activity Score 28-ESR or Clinical Disease Activity Index were not evaluated at every patient visit in the different clinics. Further studies are needed to determine whether our results are correct.

## Conclusion

We showed that the discontinuation of biologic therapy at the discharge of RA patients who had been hospitalized for infection frequently led to flares. The continuation of biologic therapy is preferable in these patients, particularly in those without risk factors for immunodeficiency such as oral steroid therapy in doses equivalent to prednisolone over 5 mg/day or chronic renal dysfunction.

## Supplementary Information

**Additional file 1: Supplementary Table S1.** Cox proportional hazards model for mortality risk after hospitalization for infection in rheumatoid arthritis patients. **Supplementary Table S2.** Types of biological DMARDs used before and after hospitalization for infection in rheumatoid arthritis patients. **Supplementary Figure S1.** Kaplan-Meier curves of RA flare-free survival stratified by biological DMARDs used after hospitalization. **Supplementary Figure S2.** Kaplan-Meier curves of hospitalized infection-free survival stratified by biological DMARDs used after hospitalization.

## Data Availability

The datasets used and/or analysed during the current study are available from the corresponding author on reasonable request.

## Abbreviations

RA: Rheumatoid arthritis
DMARDs: Biologic disease-modifying anti-rheumatic drugs
TNF: Tumour necrosis factor
RF: Rheumatoid factor
ACPA: Anti-citrullinated peptide antibody
eGFR: Estimated glomerular filtration rate
HRs: Hazard ratios
CI: Confidence intervals
