# Supplementary material for: Clinical course of patients with rheumatoid arthritis who continue or discontinue biologic therapy after hospitalization for infection: a retrospective observational study
Source: Arthritis Res Ther. 2022 Jun 1;24:131. doi: 10.1186/s13075-022-02820-y (PMC9158270; doi:10.1186/s13075-022-02820-y)
Supplement: Supplementary file 1 — Additional file 1: Supplementary Table S1. Cox proportional hazards model for mortality risk after hospitalization for infection in rheumatoid arthritis patients. Supplementary Table S2. Types of biological DMARDs used before and after hospitalization for infection in rheumatoid arthritis patients. Supplementary Figure S1. Kaplan-Meier curves of RA flare-free survival stratified by biological DMARDs used after hospitalization. Supplementary Figure S2. Kaplan-Meier curves of hospitalized infection-free survival stratified by biological DMARDs used after hospitalization. [file 13075_2022_2820_MOESM1_ESM.docx]

**Supplementary Table S1.** Cox proportional hazards model for mortality risk after hospitalization for infection in rheumatoid arthritis patients.

|  | Crude | | Model 1* | |
| --- | --- | --- | --- | --- |
|  | HR (95% CI) | *P*-value | HR (95% CI) | *P*-value |
| Age (years) | 1.06 (1.01-1.11) | 0.02 | - | - |
| Gender; female vs. male | 0.77 (0.29-2.03) | 0.60 | - | - |
| Disease duration (years) | 1.01 (0.97-1.04) | 0.70 | 1.01 (0.97-1.04) | 0.75 |
| ≥10 years vs. <10 years | 0.81 (0.33-1.99) | 0.65 | 0.81 (0.33-2.00) | 0.65 |
| Stage III or IV vs. I or II | 0.72 (0.28-1.82) | 0.48 | 0.85 (0.33-2.19) | 0.73 |
| Class III or IV vs. I or II | 3.97 (1.60-9.87) | <0.01 | 3.72 (1.48-9.39) | <0.01 |
| RF positive vs. negative | 0.54 (0.18-1.63) | 0.27 | 0.60 (0.19-1.87) | 0.37 |
| ACPA positive vs. negative | 0.63 (0.16-2.45) | 0.51 | 0.58 (0.15-2.33) | 0.45 |
| Biologics use vs. no use | 0.22 (0.08-0.58) | <0.01 | 0.26 (0.10-0.68) | <0.01 |
| MTX use vs. no use | 0.14 (0.03-0.61) | <0.01 | 0.17 (0.04-0.76) | 0.02 |
| MTX dose (mg/week) | 0.75 (0.60-0.95) | 0.02 | 0.77 (0.61-0.97) | 0.03 |
| Oral steroid, >5mg/day vs. ≤5mg/day | 2.35 (0.89-6.18) | 0.08 | 2.62 (0.96-7.20) | 0.06 |
| Oral steroid dose (mg/day) | 1.20 (1.03-1.17) | <0.01 | 1.11 (1.04-1.18) | <0.01 |
| Other DMARDs use vs. no use | 0.96 (0.35-2.71) | 0.96 | 0.84 (0.30-2.35) | 0.74 |
| Chronic lung disease | 2.05 (0.81-5.19) | 0.13 | 1.58(0.60-4.16) | 0.36 |
| Chronic renal dysfunction (eGFR <60 ml/min) | 2.08 (0.84-5.12) | 0.11 | 1.32 (0.48-3.64) | 0.59 |
| Diabetes | 2.34 (0.95-5.76) | 0.06 | 2.09 (0.83-5.25) | 0.12 |
| A history of hospital-acquired infection in the prior biologic therapy | 1.72 (0.68-4.39) | 0.25 | 1.79 (0.70-4.54) | 0.22 |

* model 1; adjusted for age and sex

Abbreviations: CI, confidence interval; HR, hazard ratio; Stage, Steinbrocker stage; Class, Steinbrocker class; RF, rheumatoid factor; ACPA, anti-citrullinated peptide antibody; MTX, methotrexate; DMARDs, disease-modifying anti-rheumatic drugs

**Supplementary Table S2.** Types of biological DMARDs used before and after hospitalization for infection in rheumatoid arthritis patients

|  | Before hospitalization  (*n* = 296) | After hospitalization  (*n* = 198) |
| --- | --- | --- |
| Infliximab, n (%) | 58 (19.6) | 32 (16.2) |
| Adalimumab, n (%) | 41 (13.9) | 24 (12.1) |
| Golimumab, n (%) | 8 (2.7) | 4 (2.0) |
| Certolizumab, n (%) | 5 (1.7) | 3 (1.5) |
| Etanercept, n (%) | 112 (37.8) | 80 (40.4) |
| Tocilizumab, n (%) | 41 (13.9) | 29 (14.6) |
| Abatacept, n (%) | 31 (10.5) | 26 (13.1) |

**Supplementary Figure S1.** Kaplan-Meier curves of RA flare-free survival stratified by biological DMARDs used after hospitalization.


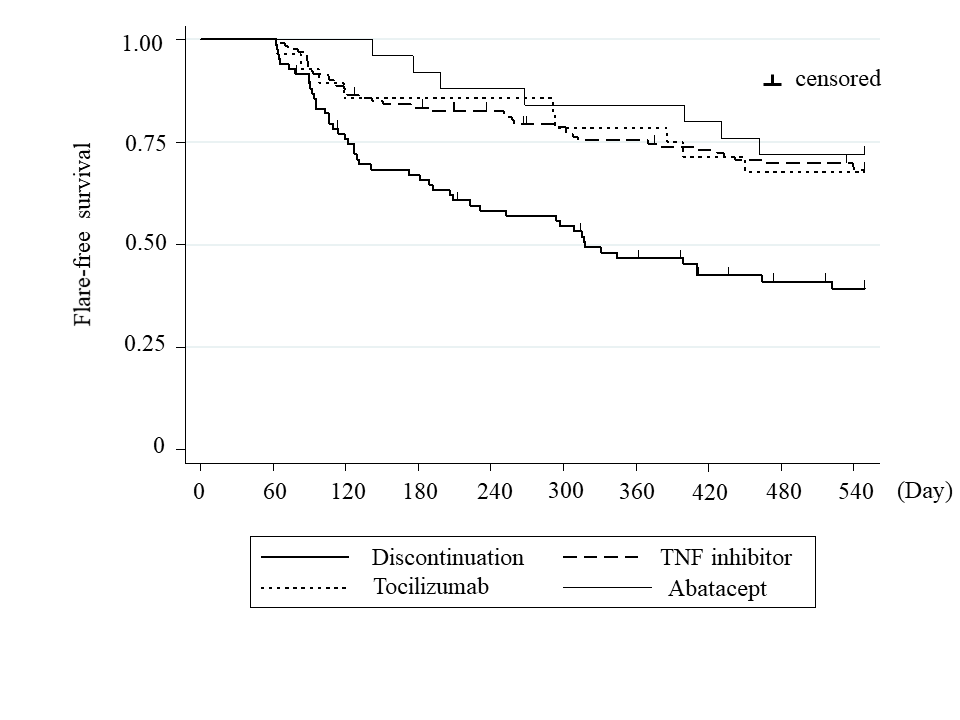


Log-rank test showed no statistically significant difference among biologic treatment groups.

**Supplementary Figure S2.** Kaplan-Meier curves of hospitalized infection-free survival stratified by biological DMARDs used after hospitalization.


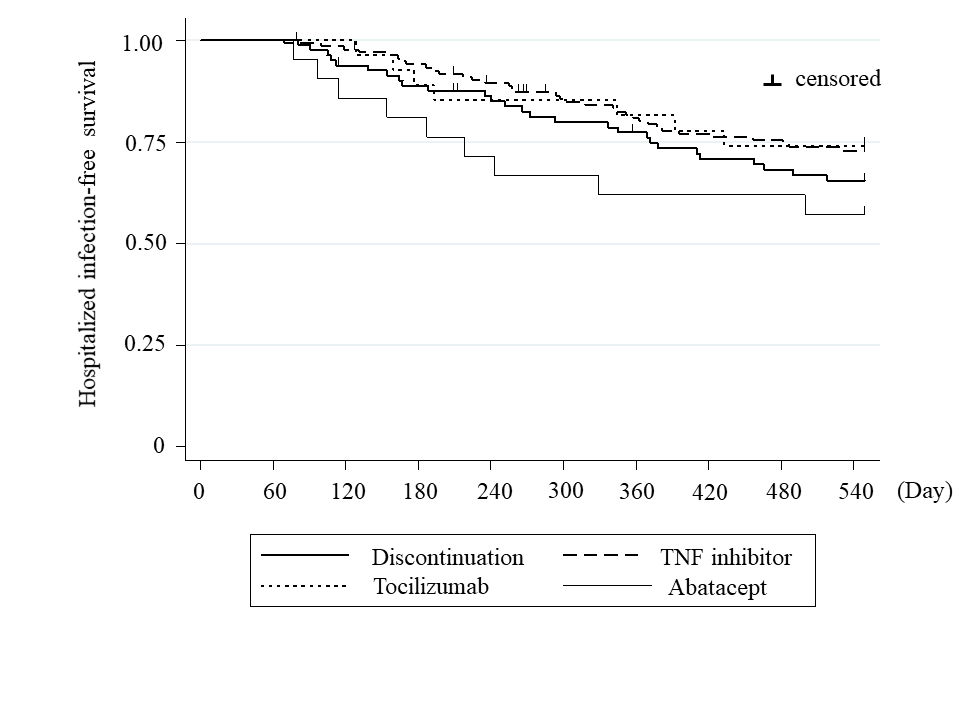


Log-rank test showed no statistically significant difference among biologic treatment groups.
